# Accessing a New Dimension in TP53 Biology: Multiplex Long Amplicon Digital PCR to Specifically Detect and Quantitate Individual *TP53* Transcripts

**DOI:** 10.3390/cancers12030769

**Published:** 2020-03-24

**Authors:** Annette Lasham, Peter Tsai, Sandra J. Fitzgerald, Sunali Y. Mehta, Nicholas S. Knowlton, Antony W. Braithwaite, Cristin G. Print

**Affiliations:** 1 Department of Molecular Medicine and Pathology, School of Medical Sciences, University of Auckland, Auckland 1142, New Zealand; p.tsai@auckland.ac.nz (P.T.); s.fitzgerald@auckland.ac.nz (S.J.F.); n.knowlton@auckland.ac.nz (N.S.K.); c.print@auckland.ac.nz (C.G.P.); 2 Maurice Wilkins Centre, University of Auckland, Auckland 1010, New Zealand; sunali.mehta@otago.ac.nz (S.Y.M.); antony.braithwaite@otago.ac.nz (A.W.B.); 3 Department of Pathology, University of Otago, Dunedin 9016, New Zealand; 4 Malaghan Institute of Medical Research, Wellington 6242, New Zealand

**Keywords:** TP53 isoforms, alternative splicing, long RNAs, digital PCR, long amplicon

## Abstract

*TP53*, the most commonly-mutated gene in cancer, undergoes complex alternative splicing. Different *TP53* transcripts play different biological roles, both in normal function and in the progression of diseases such as cancer. The study of *TP53’s* alternative RNA splice forms and their use as clinical biomarkers has been hampered by limited specificity and quantitative accuracy of current methods. *TP53* RNA splice variants differ at both 5’ and 3’ ends, but because they have a common central region of 618 bp, the individual *TP53* transcripts are impossible to specifically detect and precisely quantitate using standard PCR-based methods or short-read RNA sequencing. Therefore, we devised multiplex probe-based long amplicon droplet digital PCR (ddPCR) assays, which for the first time allow precise end-to-end quantitation of the seven major *TP53* transcripts, with amplicons ranging from 0.85 to 1.85 kb. Multiple modifications to standard ddPCR assay procedures were required to enable specific co-amplification of these long transcripts and to overcome issues with secondary structure. Using these assays, we show that several *TP53* transcripts are co-expressed in breast cancers, and illustrate the potential for this method to identify novel *TP53* transcripts in tumour cells. This capability will facilitate a new level of biological and clinical understanding of the alternatively-spliced *TP53* isoforms.

## 1. Introduction

**Figure 1:**
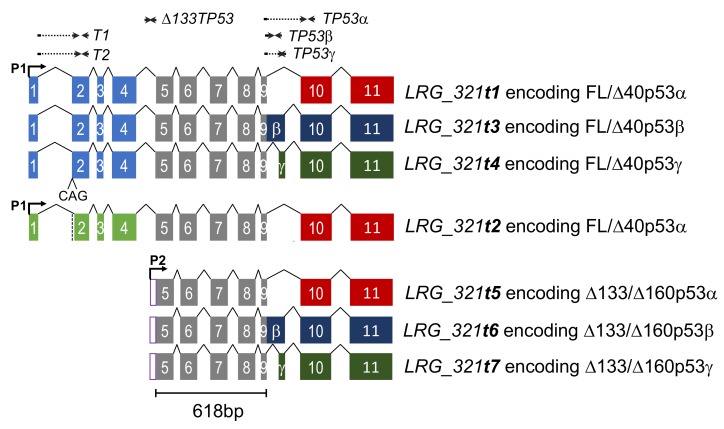
Schematic showing structure of seven *TP53* isoforms, with Ensembl transcript identifiers for the *TP53* locus (LRG_321 [7]). Transcripts *t1*-*t4* are driven off the P1 promoter, and can encode either FLp53α/β/γ or Δ40p53α/β/γ proteins. The *t2* transcript differs from the *t1* transcript by the use of an alternate acceptor splice site at the end of intron 1, leading to the deletion of a CAG at the start of exon 2 (as indicated by vertical dotted line in transcript *t2*). Transcripts *t5*–*t7* are driven off the P2 promoter and encode either Δ133p53α/β/γ or Δ160p53α/β/γ proteins. The locations of previously-published PCR-based assays to quantitate *TP53* transcript “ends” [8] are shown above, with arrows illustrating the direction of each of the primers, solid lines showing the primer sequences located within exons and dotted lines representing primer sequences that span intron/exon junctions. The exons common to all isoforms are shown in grey, and the length of this region shown below. Exons encoding *TP53α*, *TP53β* or *TP53γ* 3’ends are shown in red, blue and green, respectively.

Alternative RNA splicing increases the diversity of RNA and protein isoforms that can be expressed from a single gene, and as such this ability has been exploited by cells in natural development and in diseases, including cancer [1,2,3]. Cancer-specific splice forms can be drivers of cancer progression or can promote the hallmarks of cancer [2,3,4]. In addition to revealing biological insight, these alternate, cancer-associated RNA splice forms are potential biomarkers, but their scientific study and translation into the clinic has been hampered by limited specificity and quantitative accuracy of current methodologies [5]. An example is *TP53*, the most commonly mutated gene in cancer, which undergoes complex alternative splicing [6]. To date, the *TP53* locus has been found to express RNA transcripts encoding multiple p53 isoforms, derived from both alternative splicing and alternative promoter usage (Figure 1; [7]), with additional transcripts predicted [6,7]. Importantly, these *TP53* transcripts vary at both 5’ and 3’ ends. They share five central exons in common, which span 618 bp, therefore to determine which 5’ end and 3’ end are part of the same transcript requires amplification of >618 bp. Most PCR-based assays of *TP53* transcript abundance, including our own work, have focused on quantifying the individual transcript ends [8,9,10,11,12,13]. These studies have shown that the levels of certain 5’ or 3’ ends are significantly associated with patient prognosis in a number of cancer types, presumably reflecting the biological functions of the p53 protein sequences encoded by these alternatively-spliced RNA ends. For example, glioblastoma, colorectal or prostate cancer patients with the highest levels of the *Δ133TP53* 5’ end or the *TP53α* 3’ end have relatively poor prognosis [11,12,13]. In contrast, low levels of *TP53β* and *TP53γ* 3’ ends have been associated with poor prognosis in breast cancer [9,10]. However, until now, investigators have been uncertain which *TP53* 5’ end might be associated with which 3’ end in any one tumour or cell line, and so are unable to identify specifically which *TP53* transcripts have contributed to these clinical associations. Recently, correlations between *TP53* transcript ends have been used to infer (but without certainty) the abundance of specific *TP53* transcripts [11,12,14,15]. However, this method only provides estimates at best, and if there is low correlation, inferring which end is associated with which becomes impossible. PCR of long amplicons is one approach to resolve this problem, but to precisely quantitate specific *TP53* transcripts requires amplification of >0.8 kb products to determine the combination of “ends” that flank the shared central region [6]. Furthermore, as with other alternatively-spliced mRNAs [16], some *TP53* transcripts are prone to forming secondary structure [17,18,19], which further increases the difficulty of molecular analysis.

With the advent of digital PCR, it has become possible to amplify and quantitate single amplicons up to 905 bp using EvaGreen assays [20,21] and recently up to 600 bp in probe-based assays for detection of genome editing events [22]. Exploiting this technology, we describe for the first time a probe-based droplet digital PCR (ddPCR) approach using multiplexed probes for simultaneous quantitation of multiple 0.85–1.85 kb *TP53* splice forms in one reaction. Multiple modifications to standard ddPCR assay procedures were required to enable specific co-amplification of these long transcripts and to overcome issues with secondary structure. We also describe how this method can be easily used for the design of assays to other alternatively-spliced transcripts. With these optimised assays, researchers in the *TP53* isoform field can precisely quantitate seven major *TP53* transcripts on a whole-molecule basis. We suggest this is a significant advance over current assays that quantitate *TP53* transcript ends without knowledge of the splicing events occurring at the other end of the molecule. This methodological paper provides recipes for reliable use of these assays, illustrates their utility for different sample types and describes their development. However, the use of these assays in laboratory or clinical applications is out of scope of this paper and will be the subject of follow-on studies.

## 2. Results

### 2.1. Quantitation of TP53 t1/t3/t4 (Encoding FL/Δ40p53α/β/γ) and t5/t6/t7 (Encoding Δ133/Δ160p53α/β/γ) Transcripts

Seven major *TP53* RNA reference transcripts listed in the Ensembl database LRG_321 [7] are described in Figure 1, along with reverse transcription quantitative PCR (RT-qPCR)/ droplet digital PCR (ddPCR) assays currently used by ourselves and other investigators in the field to quantitate the 5’ or 3’ ends of these transcripts. To detect and quantitate these transcripts on a whole-molecule basis, a number of significant alterations to standard ddPCR methods were required. These are summarised in Table 1 and described in detail in Materials and Methods. 

**Figure 2:**
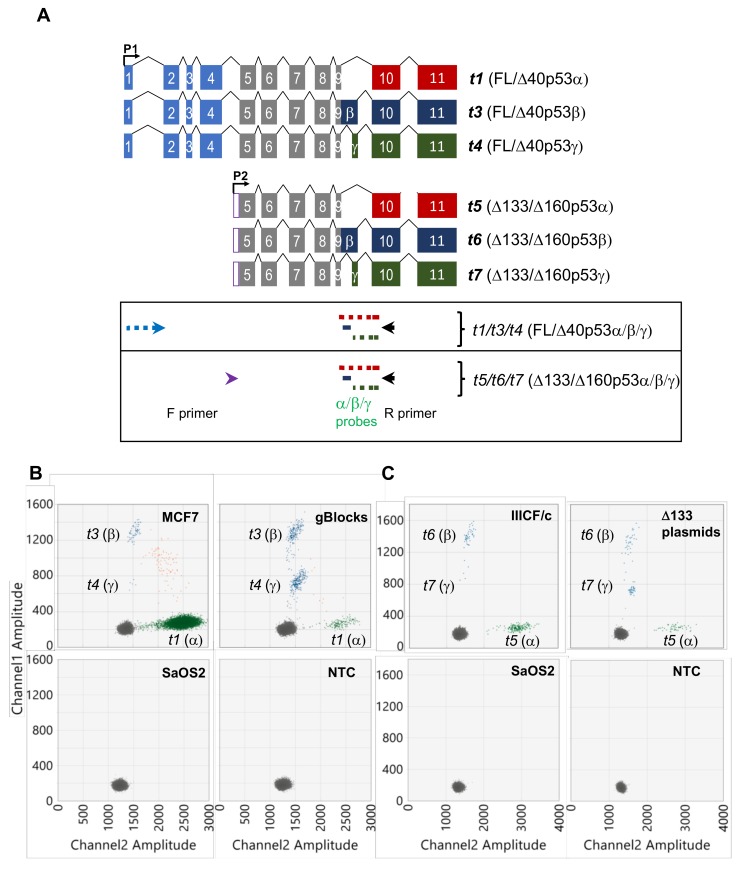
Multiplex long amplicon digital PCR assays to quantitate transcripts encoding FL/Δ40p53α/β/γ or Δ133/Δ160p53α/β/γ proteins in parallel. (**A**) Schematic showing six *TP53* transcripts, with designs of transcript-specific long amplicon ddPCR assays shown in boxes below relating to the transcripts above. Forward (F) primers, are shown as light blue and purple arrows for *t1*/*t3*/*t4* (encoding FL/Δ40p53α/β/γ) and *t5*/*t6*/*t7* (encoding Δ133/Δ160p53α/β/γ) assays, respectively. The *t1*/*t3*/*t4* assay F primer (shown in light blue) spans exons 1 and 2, including the CAG retained at the start of exon 2. The *t5*/*t6*/*t7* assay F primer (shown in purple) was designed to the Δ133p53 5’ untranslated region within intron 4. Reverse (R) primer, shown as black arrow in exon 10, was used in both assays. The red, blue and green lines represent the location of the α-, β- and γ-specific probes, respectively. The dotted lines show where a primer or probe spans an intron/exon junction. The α probe (shown in red) was designed across exons 9 and 10, the β probe (shown in dark blue) was designed within unique sequence in exon 9β, the γ probe was designed across exons 9 and 9γ. (**B**) 2-D ddPCR plots of *t1*/*t3*/*t4* (encoding FL/Δ40p53α/β/γ) assay showing the detection of each transcript in MCF7 cells. Cloned gBlocks of all *t1*/*t3*/*t4* transcripts were combined and used as a positive control. SaOS2 cells (*TP53* null) and a no-template control (NTC) are shown as negative controls. Grey dots represent droplets with no amplified product, green dots show droplets with *t1* transcript (FL/Δ40p53α) amplified, blue dots show either *t3* (FL/Δ40p53β) or *t4* (FL/Δ40p53γ) amplified as indicated, orange dots show droplets with both *t1* and *t3*, or *t1* and *t4* transcripts amplified. (**C**) 2-D ddPCR plots of *t5*/*t6*/*t7* (encoding Δ133/Δ160p53α/β/γ) assay showing the detection of each transcript in IIICF/c cells. Plasmids encoding all Δ133p53 isoforms were combined and used as a positive control. SaOS2 cells (*TP53* null) and a no-template control (NTC) are shown as negative controls. Grey dots represent droplets with no amplified product, green dots show droplets with *t5* transcript (Δ133/Δ160p53α) amplified, blue dots show either *t6* (Δ133/Δ160p53β) or *t7* (Δ133/Δ160p53γ) amplified as indicated.

Using these modifications, a multiplex long amplicon ddPCR assay was devised to measure the abundance of *t1*, *t3* and *t4* (encoding FL/Δ40p53α, β and γ) transcripts in one reaction. Following optimised parameters for primer and probe design (see Materials and Methods and Table 2), a forward primer was designed across the *TP53* exon 1 and exon 2 splice junction to specifically anneal to the 5’ end sequence present in the *t1*, *t3* and *t4* transcripts (Figure 2A and Table S1). The reverse primer was designed to a region common to all *TP53* isoforms in exon 10. For detection of the *t1* (FL/Δ40p53α) transcript, a HEX fluorophore-labelled probe was designed across the *TP53* exon 9 and 10 splice junction (Figure 2A). Detection of the *t4* (FL/Δ40p53γ) transcript was using a FAM fluorophore-labelled probe designed across the *TP53* exon 9 and 9γ splice junction (Figure 2A). For detection of the *t3* (FL/Δ40p53β) transcript, two FAM fluorophore-labelled probes were designed to sequence within *TP53* exon 9β, so that the fluorescent amplitude for the β transcript amplicon was significantly different from the γ amplicon (*t4* (FL/Δ40p53γ) transcript and Table S1). 

After optimisation of cycling conditions (see Materials and Methods), this assay was able to amplify, distinguish and quantitate the *t1*, *t3* and *t4* transcripts in cDNA derived from MCF7 breast cancer cell line RNA (Figure 2B). This showed that transcripts encoding FL/Δ40p53α, β and γ isoforms are present in these cells, with the *t1* transcript being the most abundant, and the *t4* transcript detectable, but of low abundance (the *t1* transcript approximately 80-fold and 2,600-fold more abundant than *t3* and *t4* transcripts, respectively; Figure 2B and Figure 4). The specificity of this assay was then confirmed using both positive and negative controls. Synthetic DNA fragments (gBlocks) were designed to the sequences of transcripts *t1*, *t3* and *t4*, cloned, and used in parallel (Table S2). This showed clusters of positive droplets of the same fluorescent amplitudes as observed in MCF7 cells (Figure 2B). As negative controls, cDNA from the *TP53* null sarcoma cell line, SaOS2, and a no-template control were used, which showed no *t1*, *t3* or *t4* transcripts were amplified (Figure 2B). 

Similarly, a multiplex long amplicon ddPCR assay for detection and precise quantitation of *t5*, *t6* and *t7* (encoding Δ133/Δ160p53α, β and γ) transcripts was developed (see Materials and Methods). The same reverse primer and α-, β- and γ-specific probes were used as in the *t1/t3/t4* assay above, but the forward primer was designed to a region between *TP53* exons 4 and 5 which contains the Δ133p53 transcript 5’ untranslated region (Figure 2A). Of note, this region contains a G-quadruplex structure which has been shown to regulate expression of transcripts encoding certain *TP53* isoforms [17], therefore the *Δ133TP53* forward primer was designed downstream of this structure (Table S1). After optimising cycling conditions, the assay was used to detect and quantitate all three Δ133/Δ160p53-encoding transcripts, *t5*, *t6* and *t7*, in the Li–Fraumeni cell line, IIICF/c [23] (Figure 2C). This showed that transcripts encoding Δ133/Δ160p53α, β and γ isoforms are all present in these cells, with the *t5* transcript being the most abundant, and the *t7* transcript detectable but of very low abundance (the *t5* transcript approximately 6-fold and 130-fold more abundant than *t6* and *t7* transcripts, respectively; Figure 2C and Figure 4). Plasmids encoding the sequence of each Δ133p53 isoform [24] were used as positive controls, and SaOS2 cDNA and a no template control were used as negative controls (Figure 2C). 

### 2.2. Quantitation of TP53 t1 and t2 Transcripts 

**Figure 3:**
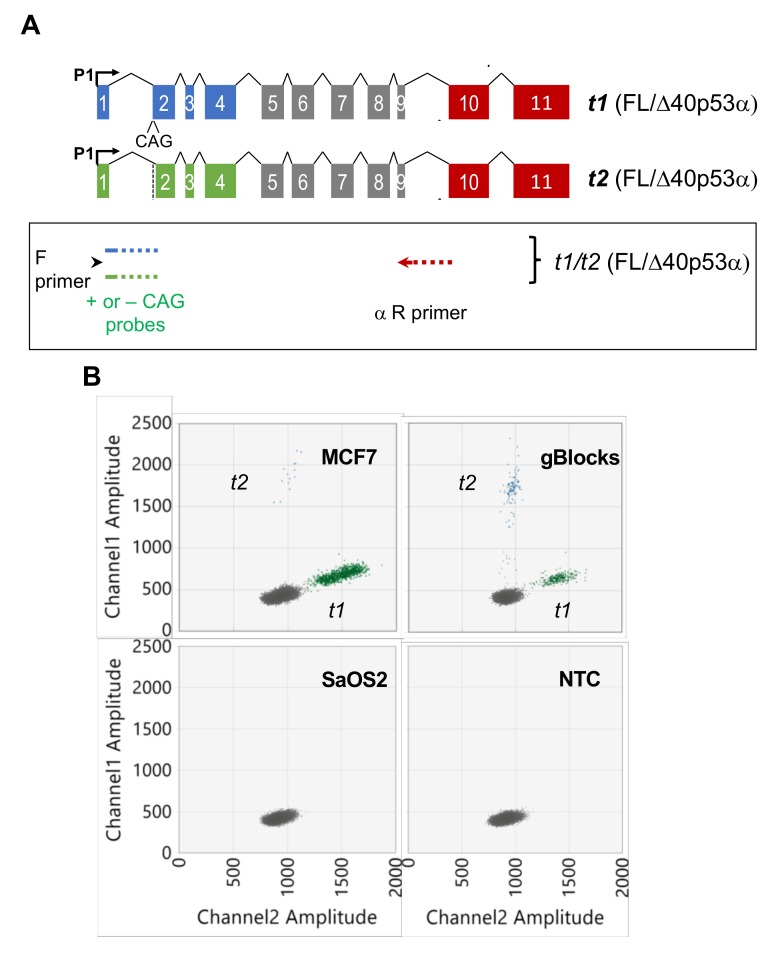
Multiplex long amplicon digital PCR method showing an entirely new assay design to quantitate *t1* and *t2* transcripts (both proposed to encode FL/Δ40p53α) in parallel. (**A**) Schematic showing *t1* and *t2* transcripts, with design of long amplicon ddPCR assay in the box below relating to the transcripts above. In this assay, probes designed across the exon 1–2 junction discriminate between transcripts with alternate 3’ splice site usage; the *t1* probe (shown in light blue) includes the CAG sequence at the start of exon 2, the *t2* probe (shown in light green) omits the CAG sequence at the start of exon 2. The dotted lines show where a primer or probe spans an intron/exon junction. Forward and reverse primers are common to both transcripts, designed to a region in exon 1 or specific for the α 3’ end sequence, respectively. P, promoter; F, forward; R, reverse. (**B**) 2-D ddPCR plots of *t1/t2* (encoding FL/Δ40p53α) assay showing the detection of each transcript in MCF7 cells. Cloned synthetic DNA fragments (gBlocks) of *t1* and *t2* transcripts were combined and used as a positive control. SaOS2 cells (*TP53* null) and a no-template control (NTC) are shown as negative controls. Grey dots represent droplets with no amplified product, green dots show droplets with *t1* transcript amplified and blue dots show amplification of *t2* transcript.

**Figure 4:**
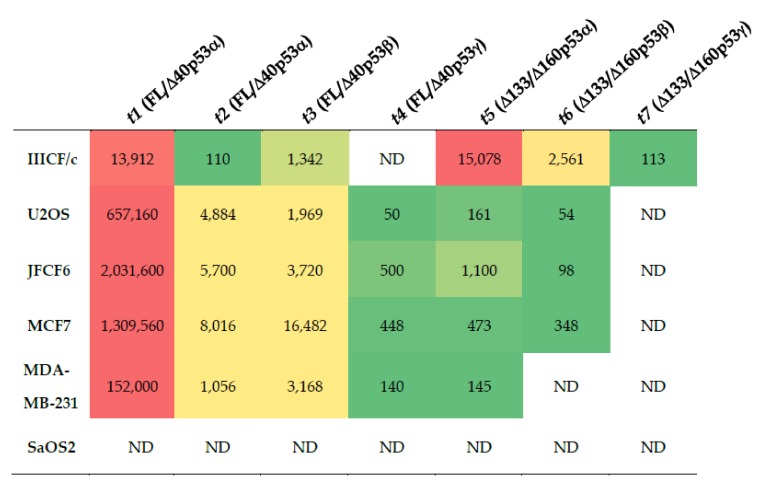
Results of multiplex long amplicon digital PCR assays showing the abundance of seven *TP53* transcripts in six cancer cell lines. Values given are copies/μg of RNA for each transcript. The relative abundance of each transcript from high to mid to low in each cell line is shown by red, yellow and green shading, respectively. LRG321 transcript identifiers are given, with encoded p53 isoforms in parentheses. ND = not detectable. Transcript abundance was ≤10 copies/μg RNA.

We next developed a separate assay to quantitate the *TP53 t1* and *t2* transcripts in one reaction. These transcripts differ by the presence or absence of a CAG at the acceptor splice site of exon 2 and are both believed to encode the FL/Δ40p53α isoform (Figure 1 [7]). These assays were devised using multiplex long amplicon ddPCR parameters similar to those described above (see Materials and Methods). In summary, the forward primer was designed to a region within *TP53* exon 1 that is common to both *t1* and *t2* transcripts and a reverse primer designed to specifically amplify the α 3’ end sequence (Figure 3A and Table S1). Probes were designed across the *TP53* exon 1 and exon 2 splice junction in order to specifically detect either *t1* or *t2* transcripts. This assay detected and precisely quantitated the *t1* and *t2* transcripts in MCF7 cells, showing that both transcripts predicted to encode the FL/Δ40p53α isoform are co-expressed (Figure 3B). The *t1* transcript was approximately 160-fold more abundant than the *t2* transcript (Figure 4). Once again, as positive controls for *t1* and *t2* transcripts, gBlocks designed to these sequences were used in parallel and showed clusters of positive droplets of the same fluorescent amplitudes as observed in MCF7 cells (Figure 3B). As negative controls, cDNA from the *TP53* null cell line, SaOS2, and a no template control were used, which showed no amplification of *t1* or *t2* transcripts (Figure 3B). 

### 2.3. Demonstration of TP53 Isoform Analysis in Cancer Cell Lines

We then used these three assays to successfully quantitate, on a whole-molecule basis, seven *TP53* transcripts in the cDNA derived from six cancer cell lines. No *TP53* transcripts were detectable in the SaOS2 cell line (Figure 4). Transcripts *t1*, *t2*, *t3* and *t5* (encoding FL/Δ40p53α, FL/Δ40p53β and Δ133/Δ160p53α) were quantifiable in all other cell lines, with *t6* (encoding Δ133/Δ160p53β) present in all except MDA-MB-231 cells where it was below the level of detectability (10 copies/μg RNA; Figure 4). The *t4* transcript (encoding FL/Δ40p53γ isoform) was expressed in all except IIICF/c cells, whereas the *t7* transcript (encoding Δ133/Δ160p53γ) was only detectable in IIICF/c cells (Figure 4). Our results show that the most abundant transcripts with a *FL/Δ40TP53_T1* or *Δ133TP53* 5’ end were *t1* (encoding FL/Δ40p53α) and *t5* (encoding Δ133/Δ160p53α), respectively (Figure 4). The most abundant transcripts with *TP53α* or *TP53β* 3’ ends were *t1* (encoding FL/Δ40p53α) and *t3* (encoding FL/Δ40p53β), respectively (Figure 4).

We have previously shown that only the most abundant *TP53* isoforms were detectable in RNA sequencing data from three of these cell lines, and that quantitation of the *TP53* transcripts using ddPCR assays of the 5’ or 3’ ends of *TP53* was significantly more sensitive and accurate, especially where the abundance of these transcripts covered a wide range [8]. Given that the long amplicon multiplex *TP53* ddPCR assays described here utilises a different method to prepare cDNA templates (see Materials and Methods), we repeated this comparison. For the same RNA preparations, transcript abundance (TPM) estimated from RNA sequencing data using the RSEM method [25] were compared to transcript abundance determined by long amplicon ddPCR (Figure S1). This suggested that for the most abundant transcripts, RNA sequencing TPM correlated well with the ddPCR results, however transcripts of lower abundance (10–1000 copies/μg RNA) were only detectable using long amplicon ddPCR. 

### 2.4. Demonstration of TP53 Isoform Analysis in Breast Cancer Patient Samples

**Figure 5:**
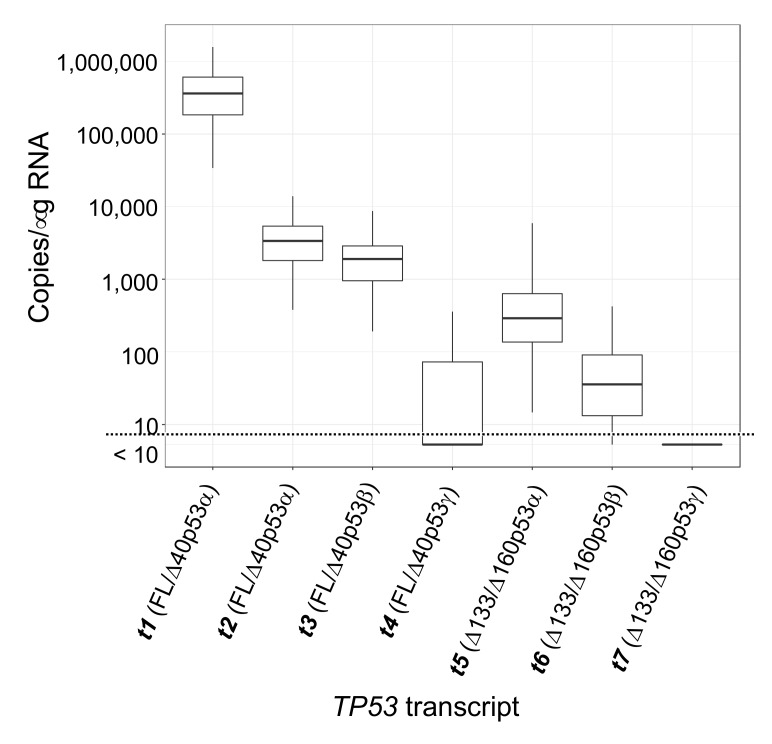
Results from multiplex long amplicon digital PCR assays applied to the detection and precise quantitation of seven *TP53* splice forms in 83 breast cancer samples. Boxplot of the number of copies/μg RNA of each *TP53* transcript in each breast cancer sample (indicated by a blue circle), showing the median, first and third quartiles, and 1.5 x interquartile range shown as whiskers. Even though different primers and probes were used, these assays allow the transcript abundance of distinct *TP53* splice forms to be directly compared. Note that the lowest level of detection is 10 copies/μg RNA, as indicated by the dotted line, so transcript levels below this cannot be quantitated.

We next measured the levels of the *TP53* transcripts in a cohort of 83 human breast tumours [26] using the ddPCR assays of *TP53* transcript 5’ and 3’ ends described previously [8] (*FL/Δ40TP53_T1, FL/Δ40TP53_T2*, *Δ133TP53 TP53* 5’ ends and the *TP53α* and *TP53β* 3’ ends; Figure S2A). We found that the levels of *FL/Δ40TP53_T1* 5’ end had the highest correlation with the *TP53α* 3’ end, and that the levels of the *Δ133TP53* 5’ end had the highest correlation with *TP53β* 3’end, consistent with other studies (Figure S2B [9,11,14,15]). The same 83 breast tumour RNA samples were then analysed using the multiplex long amplicon ddPCR assays described above, successfully quantifying the levels of the seven major *TP53* transcripts across these tumours and importantly demonstrating that these assays can be used on RNA derived from tumour samples. This analysis revealed consistent patterns of *TP53* splice variant transcript abundance across these breast tumours, with specific subgroups evident. These will be investigated in future studies and so are out of scope of this methodological paper. We observed that transcripts with a *Δ133TP53* 5’ end were predominantly *t5* and *t6* (encoding Δ133/Δ160p53α and Δ133/Δ160p53β), with median levels of 294 and 32 copies/μg RNA, respectively (Figure 5). Transcripts with *TP53β* 3’ end were *t3* and *t6* (encoding FL/Δ40p53β and Δ133/Δ160p53β), with median levels of *t3* being nearly 60-fold higher than *t6* at 1866 and 32 copies/μg RNA respectively (Figure 5). Overall the most abundant transcript in the 83 breast tumours was *t1* (encoding FL/Δ40p53α; Figure 5). Transcripts *t4* (encoding FL/Δ40p53γ) and *t7* (encoding Δ133/Δ160p53γ) were poorly or infrequently expressed and were only detectable in 22 and six tumours, respectively (Figure 5). In summary, our data suggest there are actually five *TP53* transcripts that are predominantly co-expressed in breast tumours (Figure 5). These are transcripts *t1*, *t2*, *t3*, *t5* and *t6*, encoding FL/Δ40p53α, FL/Δ40p53β, Δ133/Δ160p53α and Δ133/Δ160p53β isoforms, respectively (adjusted *p* ≤ 9.6 × 10^−11^). These results demonstrate the utility of these methods for clinical analysis and that in this cohort of breast tumours, the *TP53* transcript landscape is comprised of at least these five specific splice forms.

This analysis of 83 breast tumours provided sufficient data to compare the abundance of *TP53* transcript ends quantified by traditional RT-qPCR or ddPCR assays of *TP53* transcript ends with the abundance of entire *TP53* transcripts quantified by the whole-molecule ddPCR assays described here. Consistent with our observations above, the *FL/Δ40TP53_T1*, *FL/Δ40TP53_T2* and *Δ133TP53* 5’ ends had the highest correlation with the *t1*, *t2* and *t4* transcripts (encoding FL/Δ40p53α, FL/Δ40p53α and Δ133/Δ160p53α, respectively; Figure S2B). The *TP53α* or *TP53β* 3’ ends showed the highest correlation with *t1* (encoding FL/Δ40p53α) and *t3* (encoding FL/Δ40p53β) transcripts, respectively (Figure S2B).

### 2.5. Identification of Additional TP53 Transcript Complexity

**Figure 6:**
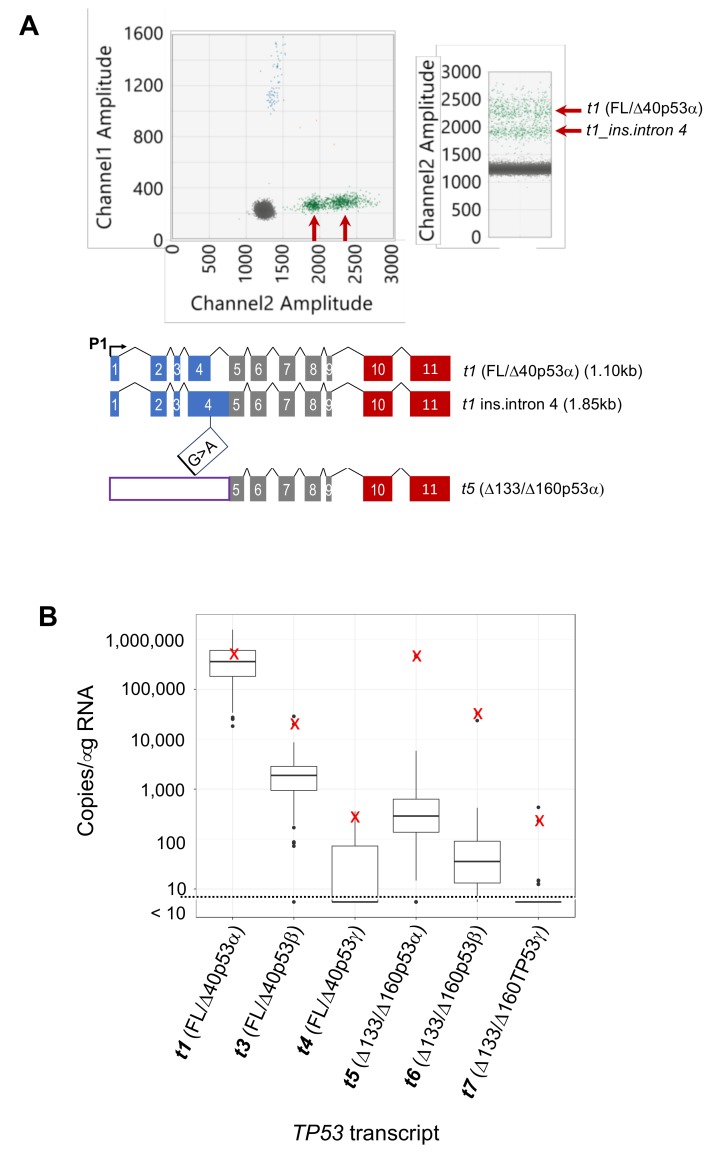
Multiplex long amplicon digital PCR *TP53 t1*/*t3*/*t4* assay showing the identification of two *t1* transcripts (encoding FL/Δ40p53α), from a mutation leading to retention of *TP53* intron 4. (**A**) Two *t1* transcripts detected in IIICF/c cells are indicated by arrows in both 2-D and 1-D ddPCR plots. The schematic below shows how the novel *t1* transcript has arisen from mutation at the donor splice site between exon 4 and intron 4 (c.375G>A), leading to the retention of intron 4. This also shows how a *t5* (Δ133/Δ160p53α) transcript can be driven off the P1 promoter. (**B**) Boxplot showing copies/μg RNA of the individual *TP53* transcripts in breast cancer cohort (as Figure 6), with **X** indicating the levels of each transcript in the tumour with the *TP53* c.375+1G>A mutation.

During our analysis, we also detected a splice variant that was initially unidentified in short read RNA sequencing analysis, and which would not be detectable using *TP53* end PCR assays. The IIICF/c cell line was generated from cells donated by a patient with Li–Fraumeni syndrome [27]. This particular Li–Fraumeni mutation (c.375G >A (COSM292894/ClinVar177825) at the *TP53* exon 4/intron 4 splice junction prevents efficient splicing and is predicted to cause loss of p53 function [28]. Interestingly, our long amplicon ddPCR analysis of this cell line identified two *t1* transcripts (Figure 6A). However, despite the presence of an in-frame stop codon, potentially making the transcript retaining the intron more susceptible to nonsense-mediated decay [29], the two *t1* transcripts appeared to be of equal abundance (amplicon sizes 1.10 kb, and with retention of intron 4 is 1.85 kb; Figure 6A and Figure S3). Based on these long amplicon ddPCR results, the RNA sequencing data of IIICF/c [8] was re-analysed using the graph-based sequence alignment program HISAT2 [30] and StringTie [31], which uses genome-guided transcriptome assembly to quantify both known and novel transcripts. This contrasts to the more commonly used RSEM method, which assigns sequencing reads only to the most ‘likely’ known transcripts. Re-analysis with HISAT2 and StringTie confirmed the presence of the two *t1* transcripts (Figure 6A). We also observed a similar result with one breast tumour sample, and confirmed by sequencing the presence of a mutation affecting the same donor splice site in intron 4 (c.375+1G>A (COSM437620/ClinVar622670); Figure S3). The transcript/s retaining the 757 bp intron would generate truncated FL/Δ40p53 proteins that do not have the DNA-binding domain, due to the presence of an in-frame stop codon 145 bp into intron 4 (Figure S3). However, these transcripts retaining the 757 bp also contain the Δ133p53 5’ untranslated region which would allow expression of the Δ133/Δ160p53 isoforms to be under control of the main (P1) promoter (Figure 6A). In support of this, the levels of the *t5*, *t6* and *t7* transcripts (encoding Δ133/Δ160p53α, Δ133/Δ160p53β and Δ133/Δ160p53γ isoforms, respectively) were similar to the *t1*, *t3* and *t4* transcripts (encoding FL/Δ40p53α, FL/Δ40p53β and FL/Δ40p53γ, respectively; Figure 6B).

## 3. Discussion

Here we describe a ddPCR method to precisely quantitate seven major *TP53* reference transcripts on a whole-molecule basis. We suggest that this method is a significant advance over current ddPCR assays that only quantitate single *TP53* transcript ends without knowledge of the splicing events occurring at the other end of the molecule. We show that this method can be applied to samples of human cancers as well as cultured cells. Therefore, this method facilitates study of the clinical consequences of specific combinations of p53 protein N- and C-termini in tumours of individual patients, in addition to analysis of cell-based laboratory studies. This methodological paper provides recipes for reliable use of these new assays, describes their development and illustrates their utility with different sample types and in different experimental or clinical applications. In particular, we illustrate this method by analysing the combination of *TP53* transcripts co-expressed in a cohort of breast tumours and in a cell line from a Li–Fraumeni patient. However, in-depth investigation of the clinical consequences of different p53 N- and C-termini combinations, made possible by this new method, including proteins encoded by the *t1* and *t2* transcripts, is out of scope of this paper and will be the subject of follow-on studies. 

Although we show the method is easy to apply to cell or tissue samples, it required substantial development due to all amplicons being longer than 0.8 kb in length and to *TP53* transcripts being prone to secondary structure formation [17,18,19]. Possibly due to these technical challenges, we believe this is the first report of a probe-based multiplex long amplicon ddPCR assay for splice variant analysis in a research setting. In order to develop these assays, we needed to make a number of critical modifications to existing digital PCR methods, including how the probes were designed. When developing these methods, we devised three assays, two using one strategy and the third of a completely novel design, which demonstrates the ease with which this method could be adapted to other genes or transcripts. Because these assays are multiplexed, they allow more information to be generated from limiting tumour samples, with the abundance of transcripts directly comparable even though different primers and probes are used, with greater sensitivity than RNA sequencing. We showed that the least abundant *TP53* transcripts (<1000 copies/μg RNA) quantitated by our long amplicon ddPCR method were undetectable in deep read RNA sequencing data. By way of illustrating the utility of these assays for clinical samples, we show that five *TP53* transcripts were co-expressed in a cohort of breast tumours, but that the *t4* and *t7* transcripts (encoding FL/Δ40p53γ and Δ133/Δ160p53γ, respectively) were only detectable in a small proportion of samples. This proof of principle screen of a cancer cohort supports the hypothesis that combinations of transcripts encoding different isoforms are co-expressed and contribute to p53’s biological and pathological roles, as emphasized in a recent publication [32]. 

We devised an assay to quantitate *t1* and *t2* transcripts (both proposed to encode FL/Δ40p53α) and observed that both were present in all breast tumours, with the *t1* transcript approximately 100-fold more abundant than the *t2*. We are uncertain of the significance of the *t2* transcript, but this assay gives researchers in the field the opportunity to quantitate this transcript in addition to *t1*. We also demonstrated how these long amplicon ddPCR assays have the potential to identify novel transcripts. Only one *TP53 t1* transcript was initially identified in RNA sequencing data from IIICF/c cells analysed by standard methods [8]. However, during the original characterization of this cell line, Northern blotting showed two *TP53* transcripts and the presence of a *TP53* splice site mutation [27]. Analysis of IIICF/c cells using our long amplicon ddPCR assays clearly detected two *t1* transcripts, which was then confirmed by performing splice-aware mapping of the RNA sequencing data. Furthermore, our assays showed that this *TP53* splicing mutation led to expression of very high levels of transcripts encoding all Δ133/Δ160p53 isoforms, and given the association of some of the Δ133p53 isoforms with invasion and metastasis [12,33], may explain why this mutation is pathogenic.

To date, the p53 field has predominantly used correlation analysis to try to draw inferential conclusions about which *TP53* transcript 5’ ends are associated with which 3’ ends [11,12,13,14,15]. Our assays now provide the ability for researchers to detect and quantitate full length *TP53* transcripts, without the need to draw inferential conclusions, and so can facilitate the understanding of *TP53* isoform biology at a more comprehensive level. In studies on *TP53* biomarkers, these long amplicon ddPCR methods could define whether a specific *TP53* transcript is prognostic without the need for inference. Where the levels of transcripts encoding particular alternate splice forms are associated with tumour classification, patient prognosis or drug response, a strength of digital PCR is that these assays can be used in a clinical setting because they allow precise quantitation of transcripts and therefore the ability to set thresholds [5,34]. Notwithstanding the above, we believe that assays of *TP53* RNA ends, as we and other use currently, remain important methodology. In at least some situations, the protein domains encoded by alternatively-spliced *TP53* transcript ends may have consistent biological and pathological functions, irrespective of splicing at the other end of the molecule. In addition, they are highly amenable to use as biomarkers in degraded, formalin-fixed paraffin-embedded (FFPE) tissues [9,10,11,12,14,33].

Notwithstanding their utility demonstrated here, there are several limitations to these long ddPCR methods. The RNA must be of high quality in order to allow amplification of (nearly) full length transcripts, therefore these assays would not be suitable for analysis of RNA derived from FFPE tissues. However, the tumour samples used in this study were all collected using standard tissue-banking methods, from which high quality RNA was obtained [26]. The assays are effectively a closed system, only allowing amplification of transcripts with specific 5’ and 3’ ends (as amplified by the forward and reverse primers), although we have demonstrated there is potential to identify novel transcripts that have these 5’ and 3’ ends but differ significantly in size from the canonical transcript. However, because the amplicons described here are >850 bp, small changes in amplicon size, for example retention of *TP53* intron 3 (109 bp), would not be clearly distinguishable. Although, if suspected, new assays of smaller amplicon size could be designed to overcome this.

The multiplex long amplicon ddPCR method described here is not only applicable to the analysis of alternatively-spliced *TP53* RNAs, but can be readily adapted to other complex loci, for example the *TP63* gene [35]. It could also have utility in the field of nonsense-mediated decay (NMD), where our methodology for example could be used to measure the effect of NMD on the *TP53* transcripts with different 3’ ends [36]. In addition, our method overcomes the limitations of standard RT-qPCR or short-read RNA sequencing methods [8] and will complement long-read RNA sequencing technologies. It can be used to determine whether there is more transcript complexity than can be inferred from short read RNA sequencing data, or as a more sensitive method to confirm and/or precisely quantitate splice forms identified by sequencing approaches [37]. It can also provide a laboratory method to corroborate inferential splice forms or splicing intermediates identified by bioinformatic and deep learning techniques [38,39]. Therefore we suggest this new method has the potential to accurately quantitate alternatively-spliced *TP53* transcripts, particularly those of lower abundance, more effectively than the short template RT-qPCR/ddPCR and RNA sequencing technologies that we and other groups in this field currently use. 

## 4. Materials and Methods 

### 4.1. Multiplex Long Amplicon ddPCR Assays

We recommend that scientists in the field use the optimised primers, probes and PCR cycling conditions detailed in Table S1, to enable detection and quantitation of seven *TP53* transcripts using three different assays. However, more detail about the development of these multiplex long amplicon ddPCR assays is described below.

#### 4.1.1. ddPCR Primer and Probe Design

Primers and probes were designed following the recommended guidelines [40] except for critical modifications shown in Table 2. 

One primer spanned a transcript-discriminating splice junction and was positioned where the 3’ end of the primer was close to the splice junction (by a few nucleotides) and yet still followed recommended guidelines, for example, GC clamping, no G at the 5’ end [40] (Table 2). The second primer was designed to detect multiple transcripts, still following guidelines [40], and in our assays this primer was designed to a common exon but close to transcript-discriminating probes (Table 2). Probes were designed with either 5’FAM or 5’HEX fluorophores, to enable multiplexing, and with double quenchers ZEN™/Iowa Black™ FQ. Where probes could not be designed to unique sequences (only *TP53β* isoforms had unique sequence), probes were designed across a different splice junction. Where possible the 5’ end of the probe was designed close to the splice junction (by a few nucleotides) and yet still followed recommended guidelines, for example, no G at 5’ end [40] (Table 2). If this was not possible, the 3’ end of the probe was positioned close to the splice junction to maintain specificity. Primers and probes were synthesised by Integrated DNA Technologies and the sequences provided in Table S1. 

#### 4.1.2. Multiplex Long Amplicon ddPCR Reactions 

For each sample, a 22 μL reaction mix was prepared consisting of 11 μL 2 x ddPCR Supermix for probes (no dUTP) (Bio-Rad), 1 μL 20 μM forward primer, 1 μL 20 μM reverse primer and 0.28 μL each 20 μM probe. DNase/RNase-free water was added to a final volume of 17 μL. To this, 5 μL dilute cDNA was added (diluted in DNase/RNase-free water at a minimum of 1:3 to a maximum of 1:100 dilution, depending on abundance of target). Reactions were mixed, briefly centrifuged for 1 min at 1000× *g* then incubated for 5 min at room temperature prior to droplet generation. Next, 20 μL of each reaction were loaded into each well of a disposable plastic cartridge (Bio-Rad, Hercules, CA, USA) with 70 μL of Droplet Generation Oil for Probes (Bio-Rad). The cartridges were placed in a QX200 droplet generator (Bio-Rad) which produced 15,000–20,000 droplets (0.89 nL/droplet). Then, 40 μL of the emulsion PCR reactions were transferred to a semi-skirted 96-well PCR plate (Eppendorf, Hamburg, Germany), and the plate sealed with tin foil for 4 s at 180 °C using the PX1 PCR plate sealer (Bio-Rad). PCR was then performed on the CX1000 Touch thermocycler (Bio-Rad), using optimised cycling conditions. The *TP53* assays described here are given in Table S1. Following PCR, the droplets from each well were read on the QX200 droplet reader (Bio-Rad) and data analysed with QuantaSoft Analysis Pro Software (version 1.0.596) according to manufacturer’s instructions. The threshold was set manually to discriminate between negative and positive droplets. cDNA was substituted with RNase-free water as a non-template control for each probe set, to allow for gating of negative droplets for analysis. The data were visualised in 1-D and 2-D plots, with the data quantitated from analysis of 2-D plots. When more than two probes were run in the same channel (e.g., for concurrent quantitation of β and γ isoforms), analysis was performed using the Advanced Classification Method in the QuantaSoft Analysis Pro Software [40]. Data from any wells with <10,000 droplets were discarded. Quantitated splice forms were calculated to copies/μg of RNA. Assay quality control was performed following dMIQE recommendations [41] (Supplementary dMIQE Information).

##### Optimisation of Cycling Conditions for the Development of New Assays 

Because of the large number of cycles and long extension times of the multiplex long amplicon ddPCR assays, initial optimisation of cycling conditions is required. We recommend (1) performing annealing temperature gradients to obtain the highest fluorescence amplitude of the positive droplets but that still retains specificity for the targets. (2) Assessing two-step (combined annealing and extension stages) versus three-step (separate annealing and extension stages) cycling conditions. In our experience, two-step cycling increased the fluorescence amplitude of the positive droplets but was more prone to “rain” (incomplete extension; data not shown). This is less of a problem for two product assays (e.g., *t1/t2* assay), but not wanted where two products are using the same fluorescent channel (e.g., for *t3* (FL/Δ40p53β) and *t4* (FL/Δ40p53γ) in *t1*/*t3*/*t4* (FL/Δ40p53α/β/γ) assays). (3) Extension times should also be evaluated, as we observed that sequences with additional predicted secondary structures required longer extension times to allow the enzyme to extend to the end of the amplicon in each cycle (data not shown). (4) Trialling amount of template used, in order to maintain specificity and provide clear separation of positive and negative droplets. (5) We also noted that the fluorescence amplitude could vary between different probe synthesis reactions, therefore new batches of probe should be evaluated initially in those assays where two amplicons are being detected in the same fluorescent channel. Note: there are newer technologies capable of detecting more than two fluorophores simultaneously, therefore multiple fluorophores could be used to allow the quantitation of many individual isoforms in the same reaction.

### 4.2. Cell Line Information 

The cell lines used were described and validated in our previous studies: IIICF/c, U2OS, JFCF6 and SaOS2 [8], MCF7 [42] and MDA-MD-231 [43]. 

### 4.3. Breast Tumour Sample Information 

Breast cancer samples were described in our previous study [26], with informed written consent obtained from all study subjects and ethical approval granted by the New Zealand Multi-Region Ethics Committee (project MEC 09/06/060). 

### 4.4. RNA Isolation and cDNA Synthesis

RNA was isolated with TRIzol reagent (Thermo Fisher, Waltham, MA, USA) according to the manufacturer’s instructions. The RNA integrity was confirmed using a 2100 Bioanalyser (Agilent, Santa Clara, CA, USA) and described in [26], and RNA concentrations determined using a NanoDrop 1000 spectrophotometer (Thermo Fisher), following the manufacturer’s instructions. The RNA samples were stored at −80 °C prior to cDNA synthesis.

For cDNA synthesis, 2 μg of RNA was DNase I treated as follows: RNA was diluted in RNase/DNase-free water in a total volume of 8.5 μL. To this, 1 μL 10x DNase I buffer (Thermo Fisher) and 0.25U DNase I (Thermo Fisher) were added and reaction incubated at room temperature for 10 min. Then 0.5 μL 25mM EDTA was added, the reaction incubated at 65 °C for 10 min followed by ice for 10 min. Next 0.5 μL 50 μM Oligo dT (Thermo Fisher), 0.5 μL 50 μM *TP53* gene-specific primer (Table S1) and 1 μL 10 mM dNTPs (Thermo Fisher) were added and the reaction incubated at 65 °C for 5 min followed by ice for 5 min. The reverse transcription reactions were set up using Superscript IV (Thermo Fisher) as follows, preparing a master mix for the number of samples plus one extra. Per sample, the reaction contained 4 μL 5x SuperScript IV buffer (Thermo Fisher), 1 μL 100 mM DTT (Thermo Fisher), 1 μL RNase/DNase-free water, 40U RNase Inhibitor (Thermo Fisher), 100U SuperScript IV Reverse Transcriptase (Thermo Fisher), in a final reaction volume of 20 μL. Reactions were incubated at 50 °C for 15 min, then 80 °C for 10 min. cDNA templates for *TP53* 5’ and 3’ end ddPCR assays were prepared using random hexamers as previously described [8]. All cDNAs were stored at −20 °C until ready for ddPCR analysis.

### 4.5. Cloning of Synthetic DNA Fragments (gBlocks) 

Based on sequences for five transcripts expressed from the *TP53* locus as described in the Ensembl database [7], gBlocks (IDT) were designed for *LRG_321t1* (encoding FL/Δ40p53α), *LRG_321t2* (also predicted to encode FL/Δ40p53α), *LRG_321t3* (encoding FL/Δ40p53β), *LRG_321t4* (encoding FL/Δ40p53γ) (Figure 1). These synthetic DNAs were designed with restriction sites for EcoRI and BamHI at the 5’ and 3’ ends, respectively (Table S2). gBlocks were digested and cleaned up using QiaQuick PCR columns (Qiagen, Hilden, Germany). Each gBlock was then ligated into pIRES2:EGFP (Clontech Laboratories, Takara Bio USA, Inc.) and transformed into DH5α competent cells. DNA from positive colonies was prepared using QIAamp DNA mini kit (Qiagen) and sequence confirmed prior to using at fg amounts in long amplicon ddPCR assays.

### 4.6. RNA-Sequence Analysis

Using the data and procedure described in [8], raw RNA sequences were quality trimmed using cutadapt v1.9.1 to remove surplus adapters, bases with Phred score < 20, and paired reads < 50 bp post-trimming. Trimmed reads were analysed using two different methods: (1) HISAT2—reads were aligned using HISAT2 [30] v2.1.0. and transcript abundance quantified using StringTie [31] v1.3.4, and (2) RSEM—reads were aligned using STAR [44] v2.5.4b and transcript abundance quantified using RSEM [25] v1.3.0. All RNA-sequence analyses were performed using the human genome version hg19 and the gene model obtained from UCSC genome browser [45]. RNA sequence was visualised using Integrative Genomics Viewer [46].

### 4.7. Statistical Analysis

Using a cut-off of 10 copies/μg RNA and assuming transcript expression as equally likely as no transcript expression, we calculated the probability of observing the number of samples expressing *TP53* transcripts via a binomial distribution for each encoded isoform.

## 5. Conclusions

In conclusion, we describe a new multiplex ddPCR method to precisely quantitate seven individual *TP53* transcripts on a whole molecule basis. This method is distinct from the RT-qPCR/ddPCR assays that we and other groups have used until now, which quantitate single *TP53* transcript ends without knowledge of the splicing events occurring at the other end of the molecule. As proof of principle, we analysed a Li–Fraumeni cell line and found that an inherited cancer-predisposing mutation causes altered *TP53* RNA splicing and transcript expression, with implications for protein function. We show that our assays are more sensitive than RNA sequencing, and in a proof of principle study show that multiple *TP53* transcripts are co-expressed in consistent patterns in a cohort of breast tumours. We also provide detailed protocols to guide others in designing their own multiplex long amplicon ddPCR assays for the quantitation of other alternatively-spliced transcripts, or any long RNAs of interest. 

## Figures and Tables

**Table 1:** Key modifications required for multiplex long amplicon droplet digital PCR (ddPCR) assays of alternatively-spliced transcripts.

	Key Assay Design Features
1.	Primers and probes designed with high melting temperatures (≥60 °C).
2.	Probe design critical—with splice site location close to 5’ end of probe where possible.
3.	Long extension times (4–6 min) with high cycle numbers (50 cycles).
4.	Two probes to one transcript can be used to allow detection of >2 transcripts in one reaction, if only two fluorophore channels available.

**Table 2:** Summary of primer and probe design strategies.

	Design	Notes
**Primer 1**	T_m_ ≥ 60 °C Designed across splice junction with 3’ end within a few nucleotides of splice junction Follows ddPCR primer design guidelines [40]	Could be forward or reverse primer
**Primer 2**	T_m_ ≥ 60 °C Designed to common sequence for multiplex reactions Follows ddPCR primer design guidelines [40]	Could be forward or reverse primer
**Probe/s**	T_m_ ≥ 62 °C Where possible, designed across splice junction with 5’ end within a few nucleotides of splice junction Follows ddPCR probe design guidelines [40] If necessary*, designed across splice junction with 3’ end within a few nucleotides of splice junction	*If not possible to design with 5’ end within a few nucleotides of splice junction and also follow ddPCR probe guidelines, then design with probe 3’ end within a few nucleotides of splice junction. This not necessary if splice form has unique sequence.

## Supplementary Materials

The following are available online at https://www.mdpi.com/2072-6694/12/3/769/s1, Figure S1: Scatterplot showing correlation of TP53 transcript abundance from RNA sequencing and from long amplicon ddPCR; Figure S2: Analysis of TP53 transcript levels in breast tumour cohort; Figure S3: Sequence of a region of the TP53 gene, from exon 4 to exon 5; Supplementary dMIQE Information: dMIQE information as recommended by [41]; Table S1: Sequences and locations of probes and primers, and cycling conditions used for *TP53* multiplex long amplicon ddPCR assays; Table S2: Sequence of gBlocks used for cloning as positive control for *TP53* transcripts.
