# Supplementary material for: Accessing a New Dimension in TP53 Biology: Multiplex Long Amplicon Digital PCR to Specifically Detect and Quantitate Individual TP53 Transcripts
Source: Cancers (Basel). 2020 Mar 24;12(3):769. doi: 10.3390/cancers12030769 (PMC7140069; doi:10.3390/cancers12030769)
Supplement: Supplementary file 1 [file cancers-12-00769-s001.zip › Supplement Tables.pdf]

Table 1. Sequences and locations of probes and primers, and cycling conditions used for *TP53* splice form quantification by multiplex long amplicon ddPCR.

Assay	Oligonucleotide (T_m)	Location	Sequence (5′- 3′)
**Detection of *TP53* transcripts *t1/t3/4*** (encoding FL/Δ40p53α/β/γ)<br><br>(LRG_321t1/t3 and t4 transcripts. NCBI reference sequences for these are- NM_000546.5/ NM_001126114.2/ NM_001126113.2 respectively)<br><br>Amplicon sizes = 1099, 1232 and 1,159bp respectively<br><br>Cycling conditions:	Forward primer (62.0°C)	Exon 2	CTGGATTGGCAGCCAGACT
	Reverse primer (60.1°C)	Exon 10	CTGGGCATCCTTGAGTTCC
	α probe_HEX (64.9°C)	Exons 9/10	5HEX_CGGATCTGAAGGGTGAAATATTCTCCA_3IABkFQ
	β probe_FAM_1 (62.2°C)	Exon 9β	56-FAM_ACTTTGCCTGATACAGATGCTACT_3IABkFQ
	β probe_FAM_2* (64.6°C)	Exon 9β	56-FAM_TCTGTATCAGGCAAAGTCATAGAACCAT_3IABkFQ
	γ probe_FAM (64.6°C)	Exons 9/9γ	56-FAM_AGCATCTGAAGGGTGAAATATTCTCCA_3IABkFQ
	94°C for 10 min, then 50 cycles of 94°C for 30 sec, 64°C for 1 min, 72°C for 6 min, followed by 98°C for 10 min and then hold temperature of 12°C.		
**Detection of *TP53* transcripts *t5/t6/t7*** (encoding Δ133/Δ160p53α/β/γ)<br><br>(LRG_321t5/t6 and t7 transcripts. NCBI reference sequences for these are- NM_001126115.1/ NM_001126116.1 NM_0011261137.1)<br><br>Amplicon sizes = 858, 991 and 918bp respectively<br><br>Cycling conditions:	Forward primer (60.6°C)	Intron 4	CCTGACTTTCAACTCTGTCTCC
	Reverse primer (60.1°C)	Exon 10	CTGGGCATCCTTGAGTTCC
	α probe_HEX (64.9°C)	Exons 9/10	5HEX_CGGATCTGAAGGGTGAAATATTCTCCA_3IABkFQ
	β probe_FAM_1 (62.2°C)	Exon 9β	56-FAM_ACTTTGCCTGATACAGATGCTACT_3IABkFQ
	β probe_FAM_2* (64.6°C)	Exon 9β	56-FAM_TCTGTATCAGGCAAAGTCATAGAACCAT_3IABkFQ
	γ probe_FAM (64.6°C)	Exons 9/9γ	56-FAM_AGCATCTGAAGGGTGAAATATTCTCCA_3IABkFQ
	94°C for 10 min, then 50 cycles of 94°C for 30 sec, 64°C for 1 min, 72°C for 6 min, followed by 98°C for 10 min and then hold temperature of 12°C.		
**Detection of *TP53* transcripts *t1* and *t2*** (both transcripts predicted to encode FL/Δ40p53α)<br><br>(LRG_321t1 and t2 transcripts. NCBI reference sequences for these are- NM_000546.5/ NM_001126112.2)	Forward primer (62.5°C)	Exon 1	TGGGAGCGTGCTTTCCAC
	Reverse primer (62.0°C)	Exons 9/10	CCCACGGATCTGAAGGGTG
	t1 probe_HEX (65.2°C)	Exon 1/2	5HEX_CCGGAAGGCAGTCTGGCTG_3IABkFQ

Assay	Oligonucleotide (T_m)	Location	Sequence (5′- 3′)
Cycling conditions:<br><br>Amplicon sizes = 1075 and 1072bp respectively.	t2 probe_FAM (64.3°C)	Exon 1/2	56-FAM_CGGAAGGCAGTCTGGCCA_3IABkFQ
	94°C for 10 min, then 50 cycles of 94°C for 30 sec, 62°C for 1 min, 72°C for 6 min, followed by 98°C for 10 min and then hold temperature of 12°C.		
	**Oligonucleotide**	**Location**	**Sequence (5′- 3′)**
**cDNA synthesis**	*TP53* gene-specific primer	Exon 11	AAAGACCCAAAACCCAAAATG

*Reactions can be run as and , and , or , and , with concurrent detection and quantitation of and isoforms possible in the FAM channel by using two probes.

Table S2. Sequences of gBlocks used for cloning as positive controls for *TP53* transcripts. Lowercase font indicates sequence for restriction sites appended to *TP53* sequences

***TP53* transcript**	**Sequence of gBlocks**
LRG321_t1 (encoding FL/Δ40p53α)<br><br>(NCBI Reference Sequence NM_000546.5)	ccggaattcGCGTTCGGGCTGGGAGCGTGCTTTCCACGACGGTGACACGCTTCCCTGGATTG<br>GCAGCCAGACTGCCTTCCGGGTCACTGCCATGGAGGAGCCGCAGTCAGATCCTAGCGTC<br>GAGCCCCCTCTGAGTCAGGAAACATTTTCAGACCTATGGAAACTACTTCCTGAAAACAACG<br>TTCTGTCCCCCTTGCCGTCCCAAGCAATGGATGATTTGATGCTGTCCCCGGACGATATTGA<br>ACAATGGTTCACTGAAGACCCAGGTCCAGATGAAGCTCCCAGAATGCCAGAGGCTGCTCC<br>CCCCGTGGCCCCTGCACCAGCAGCTCCTACACCGGCGGCCCCTGCACCAGCCCCCTCCT<br>GGCCCCTGTCATCTTCTGTCCCTTCCCAGAAAACCTACCAGGGCAGCTACGGTTTCCGTCT<br>GGGCTTCTTGCATTCTGGGACAGCCAAGTCTGTGACTTGCACGTACTCCCCTGCCCTCAAC<br>AAGATGTTTTGCCAACTGGCCAAGACCTGCCCTGTGCAGCTGTGGGTTGATTCCACACCCC<br>CGCCCGGCACCCGCGTCCGCGCCATGGCCATCTACAAGCAGTCACAGCACATGACGGAG<br>GTTGTGAGGCGCTGCCCCCACCATGAGCGCTGCTCAGATAGCGATGGTCTGGCCCCTCCT<br>CAGCATCTTATCCGAGTGGAAGGAAATTTGCGTGTGGAGTATTTGGATGACAGAAACACTT<br>TTCGACATAGTGTGGTGGTGCCCTATGAGCCGCCTGAGGTTGGCTCTGACTGTACCACCA<br>TCCACTACAACTACATGTGTAACAGTTCCTGCATGGGCGGCATGAACCGGAGGCCCATCCT<br>CACCATCATCACACTGGAAGACTCCAGTGGTAATCTACTGGGACGGAACAGCTTTGAGGTG<br>CGTGTTTGTGCCTGTCCTGGGAGAGACCGGCGCACAGAGGAAGAGAATCTCCGCAAGAAA<br>GGGGAGCCTCACCACGAGCTGCCCCCAGGGAGCACTAAGCGAGCACTGCCCAACAACAC<br>CAGCTCCTCTCCCCAGCCAAAGAAGAAACCACTGGATGGAGAATATTTCACCCTTCAGATC<br>CGTGGGCGTGAGCGCTTCGAGATGTTCCGAGAGCTGAATGAGGCCTTGGAACTCAAGGAT<br>GCCCAGGCTGGGAAGGAGCCAGGGGGGAGCAGGGCTCACTCCAGCCACCTGAAGTCCAA<br>AAAGGGTCAGTCTACCTCCCGCCATAAAAAACTCATGTTCAAGACAGAAGGGCCTGACTCA<br>GACTGACATTCTCCACTTCTTGTTCCCCACTGACAGCCTCCCggatccgcg
LRG321_t3 (encoding FL/Δ40p53β)<br><br>(NCBI Reference Sequence NM_001126114.2)	ccggaattcGCGTTCGGGCTGGGAGCGTGCTTTCCACGACGGTGACACGCTTCCCTGGATTG<br>GCAGCCAGACTGCCTTCCGGGTCACTGCCATGGAGGAGCCGCAGTCAGATCCTAGCGTC<br>GAGCCCCCTCTGAGTCAGGAAACATTTTCAGACCTATGGAAACTACTTCCTGAAAACAACG<br>TTCTGTCCCCCTTGCCGTCCCAAGCAATGGATGATTTGATGCTGTCCCCGGACGATATTGA<br>ACAATGGTTCACTGAAGACCCAGGTCCAGATGAAGCTCCCAGAATGCCAGAGGCTGCTCC<br>CCCCGTGGCCCCTGCACCAGCAGCTCCTACACCGGCGGCCCCTGCACCAGCCCCCTCCT<br>GGCCCCTGTCATCTTCTGTCCCTTCCCAGAAAACCTACCAGGGCAGCTACGGTTTCCGTCT<br>GGGCTTCTTGCATTCTGGGACAGCCAAGTCTGTGACTTGCACGTACTCCCCTGCCCTCAAC<br>AAGATGTTTTGCCAACTGGCCAAGACCTGCCCTGTGCAGCTGTGGGTTGATTCCACACCCC<br>CGCCCGGCACCCGCGTCCGCGCCATGGCCATCTACAAGCAGTCACAGCACATGACGGAG<br>GTTGTGAGGCGCTGCCCCCACCATGAGCGCTGCTCAGATAGCGATGGTCTGGCCCCTCCT<br>CAGCATCTTATCCGAGTGGAAGGAAATTTGCGTGTGGAGTATTTGGATGACAGAAACACTT<br>TTCGACATAGTGTGGTGGTGCCCTATGAGCCGCCTGAGGTTGGCTCTGACTGTACCACCA<br>TCCACTACAACTACATGTGTAACAGTTCCTGCATGGGCGGCATGAACCGGAGGCCCATCCT<br>CACCATCATCACACTGGAAGACTCCAGTGGTAATCTACTGGGACGGAACAGCTTTGAGGTG<br>CGTGTTTGTGCCTGTCCTGGGAGAGACCGGCGCACAGAGGAAGAGAATCTCCGCAAGAAA<br>GGGGAGCCTCACCACGAGCTGCCCCCAGGGAGCACTAAGCGAGCACTGCCCAACAACAC<br>CAGCTCCTCTCCCCAGCCAAAGAAGAAACCACTGGATGGAGAATATTTCACCCTTCAGGAC<br>CAGACCAGCTTTCAAAAAGAAAATTGTTAAAGAGAGCATGAAAATGGTTCTATGACTTTGCC<br>TGATACAGATGCTACTTGACTTACGATGGTGTTACTTCCTGATAAACTCGTCGTAAGTTGAA<br>AATATTATCCGTGGGCGTGAGCGCTTCGAGATGTTCCGAGAGCTGAATGAGGCCTTGGAA<br>CTCAAGGATGCCCAGGCTGGGAAGGAGCCAGGGGGGAGCAGGGCTCACTCCAGCCACCT<br>GAAGTCCAAAAggatccgcg
LRG321_t4 (encoding FL/Δ40p53γ)<br><br>(NCBI Reference Sequence NM_001126113.2)	ccggaattcGCGTTCGGGCTGGGAGCGTGCTTTCCACGACGGTGACACGCTTCCCTGGATTG<br>GCAGCCAGACTGCCTTCCGGGTCACTGCCatgGAGGAGCCGCAGTCAGATCCTAGCGTCG<br>AGCCCCCTCTGAGTCAGGAAACATTTTCAGACCTATGGAAACTACTTCCTGAAAACAACGTT<br>CTGTCCCCCTTGCCGTCCCAAGCAatgGATGATTTGATGCTGTCCCCGGACGATATTGAACA<br>ATGGTTCACTGAAGACCCAGGTCCAGATGAAGCTCCCAGAATGCCAGAGGCTGCTCCCCC<br>CGTGGCCCCTGCACCAGCAGCTCCTACACCGGCGGCCCCTGCACCAGCCCCCTCCTGGC<br>CCCTGTCATCTTCTGTCCCTTCCCAGAAAACCTACCAGGGCAGCTACGGTTTCCGTCTGGG<br>CTTCTTGCATTCTGGGACAGCCAAGTCTGTGACTTGCACGTACTCCCCTGCCCTCAACAAG<br>ATGTTTTGCCAACTGGCCAAGACCTGCCCTGTGCAGCTGTGGGTTGATTCCACACCCCCG<br>CCCGGCACCCGCGTCCGCGCCATGGCCATCTACAAGCAGTCACAGCACATGACGGAGGT<br>TGTGAGGCGCTGCCCCCACCATGAGCGCTGCTCAGATAGCGATGGTCTGGCCCCTCCTCA<br>GCATCTTATCCGAGTGGAAGGAAATTTGCGTGTGGAGTATTTGGATGACAGAAACACTTTT<br>CGACATAGTGTGGTGGTGCCCTATGAGCCGCCTGAGGTTGGCTCTGACTGTACCACCATC<br>CACTACAACTACATGTGTAACAGTTCCTGCATGGGCGGCATGAACCGGAGGCCCATCCTC<br>ACCATCATCACACTGGAAGACTCCAGTGGTAATCTACTGGGACGGAACAGCTTTGAGGTGC<br>GTGTTTGTGCCTGTCCTGGGAGAGACCGGCGCACAGAGGAAGAGAATCTCCGCAAGAAAG<br>GGGAGCCTCACCACGAGCTGCCCCCAGGGAGCACTAAGCGAGCACTGCCCAACAACACC<br>AGCTCCTCTCCCCAGCCAAAGAAGAAACCACTGGATGGAGAATATTTCACCCTTCAGATGC<br>TACTTGACTTACGATGGTGTTACTTCCTGATAAACTCGTCGTAAGTTGAAAATATTATCCGT<br>GGGCGTGAGCGCTTCGAGATGTTCCGAGAGCTGAATGAGGCCTTGGAACTCAAGGATGCC<br>CAGGCTGGGAAGGAGCCAGGGGGGAGCAGGGCTCACTCCAGCCACCTGAAGTCCAAAAg<br>gatccgcg
LRG321_t2 (encoding FL/Δ40p53α)	ccggaattcGCGTTCGGGCTGGGAGCGTGCTTTCCACGACGGTGACACGCTTCCCTGGATTG<br>GCCAGACTGCCTTCCGGGTCACTGCCatgGAGGAGCCGCAGTCAGATCCTAGCGTCGAGC<br>CCCCTCTGAGTCAGGAAACATTTTCAGACCTATGGAAACTACTTCCTGAAAACAACGTTCTG

(NCBI Reference Sequence NM_001126112.2)	TCCCCCTTGCCGTCCCAAGCAatgGATGATTTGATGCTGTCCCCGGACGATATTGAACAATG<br>GTTCACTGAAGACCCAGGTCCAGATGAAGCTCCCAGAATGCCAGAGGCTGCTCCCCCCGT<br>GGCCCCTGCACCAGCAGCTCCTACACCGGCGGCCCCTGCACCAGCCCCCTCCTGGCCCC<br>TGTCATCTTCTGTCCCTTCCCAGAAAACCTACCAGGGCAGCTACGGTTTCCGTCTGGGCTT<br>CTTGCATTCTGGGACAGCCAAGTCTGTGACTTGCACGTACTCCCCTGCCCTCAACAAGATG<br>TTTTGCCAACTGGCCAAGACCTGCCCTGTGCAGCTGTGGGTTGATTCCACACCCCCGCCC<br>GGCACCCGCGTCCGCGCCATGGCCATCTACAAGCAGTCACAGCACATGACGGAGGTTGT<br>GAGGCGCTGCCCCCACCATGAGCGCTGCTCAGATAGCGATGGTCTGGCCCCTCCTCAGC<br>ATCTTATCCGAGTGGAAGGAAATTTGCGTGTGGAGTATTTGGATGACAGAAACACTTTTCG<br>ACATAGTGTGGTGGTGCCCTATGAGCCGCCTGAGGTTGGCTCTGACTGTACCACCATCCA<br>CTACAACTACATGTGTAACAGTTCCTGCATGGGCGGCATGAACCGGAGGCCCATCCTCAC<br>CATCATCACACTGGAAGACTCCAGTGGTAATCTACTGGGACGGAACAGCTTTGAGGTGCGT<br>GTTTGTGCCTGTCCTGGGAGAGACCGGCGCACAGAGGAAGAGAATCTCCGCAAGAAAGG<br>GGAGCCTCACCACGAGCTGCCCCCAGGGAGCACTAAGCGAGCACTGCCCAACAACACCA<br>GCTCCTCTCCCCAGCCAAAGAAGAAACCACTGGATGGAGAATATTTCACCCTTCAGATCCG<br>TGGGCGTGAGCGCTTCGAGATGTTCCGAGAGCTGAATGAGGCCTTGGAACTCAAGGATGC<br>CCAGGCTGGGAAGGAGCCAGGGGGGAGCAGGGCTCACTCCAGCCACCTGAAGTCCAAAA<br>AGGGTCAGTCTACCTCCCGCCATAAAAAACTCATGTTCAAGACAGAAGGGCCTGACTCAGA<br>CTGACATTCTCCACTTCTTGTTCCCCACTGACAGCCTCCCggatccgcg
