# Supplementary material for: Accessing a New Dimension in TP53 Biology: Multiplex Long Amplicon Digital PCR to Specifically Detect and Quantitate Individual TP53 Transcripts
Source: Cancers (Basel). 2020 Mar 24;12(3):769. doi: 10.3390/cancers12030769 (PMC7140069; doi:10.3390/cancers12030769)
Supplement: Supplementary file 1 [file cancers-12-00769-s001.zip › Supplementary Figures.pdf]

Figure S1

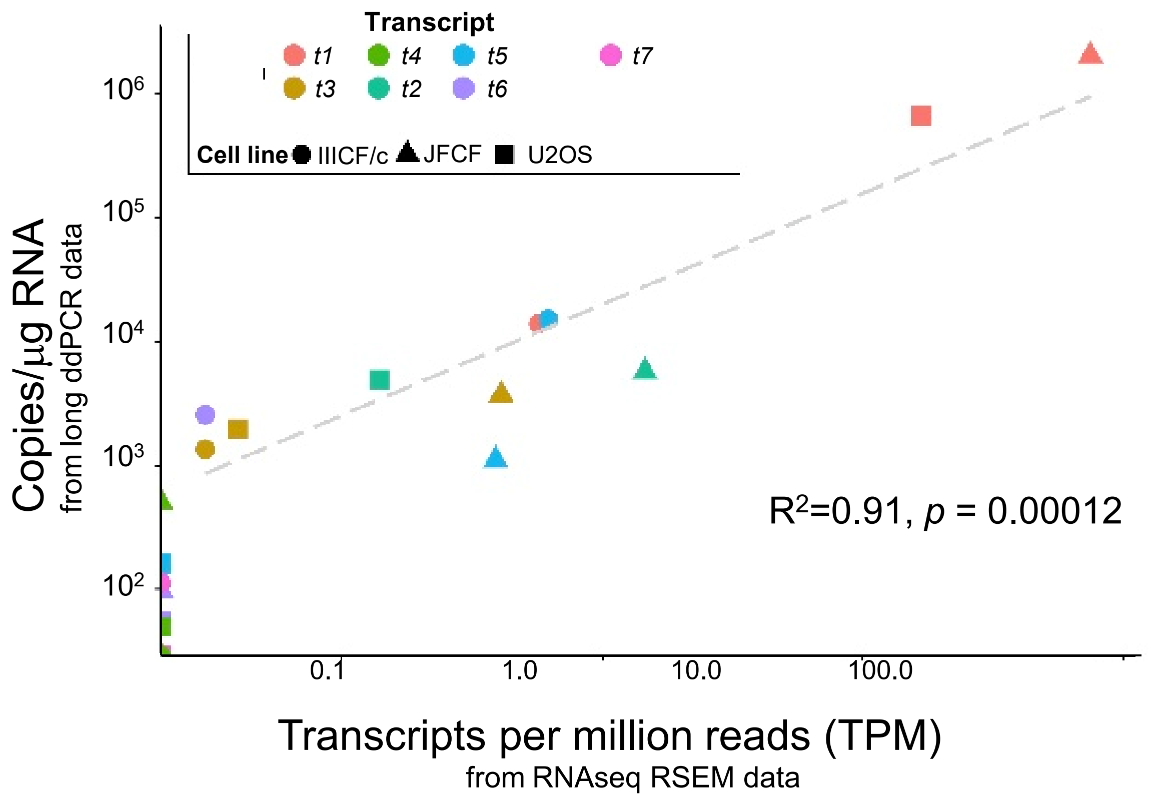

Figure S1.
Scatterplot showing correlation of *TP53* transcript abundance determined by two methods; RSEM data from RNA sequencing (Mehta *et al*., Cancer Res 2016) and copies/µg RNA from long amplicon ddPCR, demonstrating that expression levels of *TP53* transcripts determined by RNA sequencing data and long amplicon ddPCR shows good correlation for transcripts expressed above ~ 1,000 copies/µg RNA. Below this level, *TP53* transcript expression is undetectable by RNA sequencing. Data for each cell line is shown by symbol (● = IIICF/c, ▲ = JFCF, ■ = U2OS) and each *TP53* transcript by colour- ***t1*** (encoding FL/Δ40p53α), ***t2*** (encoding FL/Δ40p53α), ***t3*** (encoding FL/Δ40p53β), ***t4*** (encoding FL/Δ40p53γ), ***t5*** (encoding Δ133/Δ160p53α), ***t6*** (encoding Δ133/Δ160p53β) and ***t7*** (encoding Δ133/Δ160p53γ).

Figure S2

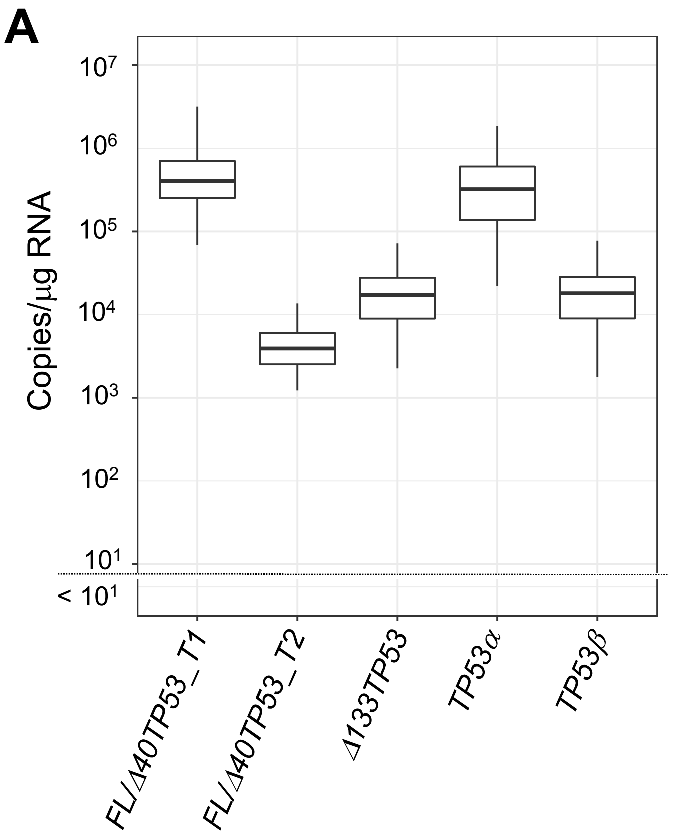

B

	FL/Δ40TP53_T1	*FL/Δ40TP53_T2*	*Δ133TP53*	*TP53α*	*TP53β*	*t1* (FL/Δ40p53α)	*t2* (FL/Δ40p53α)	*t3* (FL/Δ40p53β)	*t4* (FL/Δ40p53γ)	*t5* (Δ133/Δ160p53α)	*t6* (Δ133/Δ160p53β)	*t7* (Δ133/Δ160p53γ)
FL/Δ40TP53_T1	1.00	0.93	0.59	0.68	0.47	0.69	0.67	0.32	0.20	0.46	0.17	0.22
FL/Δ40TP53_T2	0.93	1.00	0.54	0.78	0.51	0.68	0.66	0.25	-0.11	0.48	0.19	0.18
Δ133TP53	0.59	0.54	1.00	0.30	0.53	0.27	0.25	0.25	0.20	0.52	0.37	0.24
TP53α	0.68	0.78	0.30	1.00	0.75	0.66	0.62	0.39	-0.01	0.33	0.26	0.13
TP53β	0.47	0.51	0.53	0.75	1.00	0.40	0.35	0.44	0.04	0.37	0.38	0.10
***t1* (FL/Δ40p53α)**	0.69	0.68	0.27	0.66	0.40	1.00	0.98	0.65	0.24	0.52	0.33	0.20
***t2* (FL/Δ40p53α)**	0.67	0.66	0.25	0.62	0.35	0.98	1.00	0.66	0.22	0.51	0.32	0.20
***t3* (FL/Δ40p53β)**	0.32	0.25	0.25	0.39	0.44	0.65	0.66	1.00	0.36	0.54	0.62	0.22
***t4* (FL/Δ40p53γ)**	0.20	-0.11	0.20	-0.01	0.04	0.24	0.22	0.36	1.00	0.27	0.19	0.26
***t5* (Δ133p53α)**	0.46	0.48	0.52	0.33	0.37	0.52	0.51	0.54	0.27	1.00	0.66	0.29
***t6* (Δ133p53β)**	0.17	0.19	0.37	0.26	0.38	0.33	0.32	0.62	0.19	0.66	1.00	0.33
***t7* (Δ133p53γ)**	0.22	0.18	0.24	0.13	0.10	0.20	0.20	0.22	0.26	0.29	0.33	1.00

Figure S2.
Analysis of *TP53* expression in breast tumour cohort. **A.** Boxplot showing copies/µg RNA for each *TP53* 5' (*FL/Δ40TP53_T1, T2 or Δ133TP53*) or 3' (*TP53α or β*) end. **B.** Results of correlation analysis of the expression levels of the *TP53* transcript ends and full length *TP53* transcripts. Values shown are Spearman's ρ.

Figure S3

7579577 CCCAAGCAAT GGATGATTTG ATGCTGTCCC CGGACGATAT TGAACAATGG TTCACTGAAG *exon 4*

7579517 ACCCAGGTCC AGATGAAGCT CCCAGAATGC CAGAGGCTGC TCCCCCCGTG GCCCCTGCAC

7579457 CAGCAGCTCC TACACCGGCG GCCCCTGCAC CAGCCCCCTC CTGGCCCCTG TCATCTTCTG

7579397 TCCCTTCCCA GAAAACCTAC CAGGGCAGCT ACGGTTTCCG TCTGGGCTTC TTGCATTCTG

7579337 GGACAGCCAA GTCTGTGACT TGCAC**AA**TCA GTTGCCCTGA GGGGCTGGCT TCCATGAGAC *intron 4*

7579277 TTCAATGCCT GGCCGTATCC CCCTGCATTT CTTTTGTTTG GAACTTTGGG ATTCCTCTTC

7579217 ACCCTTTGGC TTCCTGTCAG TGTTTTTTTA TAGTTTACCC ACTTAATGTG *TGA*tctctga

7579157 ctcctgtccc aaagttgaat attccccccct tgaatttggg cttttatcca tcccatcaca

7579097 ccctcagcat ctctcctggg gatgcagaac ttttcttttt cttcatccac gtgtattcct

7579037 tggcttttga aaataagctc ctgaccaggc ttggtggctc acacctgcaa tcccagcact

7578977 ctcaaagagg ccaaggcagg cagatcacct gagcccagga gttcaagacc agcctgggta

7578917 acatgatgaa acctcgtctc tacaaaaaaa tacaaaaaat tagccaggca tggtggtgca

7578857 cacctatagt cccagccact taggaggctg aggtgggaag atcact**tgag** **gccaggagat**

7578797 **ggaggctgca** **gtgagctgtg** **atcacaccac** **tgtgctccag** **cctgagtgac** **agagcaagac**

7578737 **cctatctcaa** **aaaaaaaaaa** **aaaaaagaaa** **agctcctgag** **gtgtagacgc** **caactctctc**

7578677 **tagctcgcta** **gtgggttgca** **ggaggtgctt** **acgcatgttt** **gtttctttgc** **tgccgtcttc**

7578617 **cagttgcttt** **atctgttcac** **ttgtgccctg** **actttcaact** **ctgtctcctt** **cctcttccta**

7578557 **cag**TACTCCC CTGCCCTCAA CAAGATGTTT TGCCAACTGG CCAAGACCTG CCCTGTGCAG *exon 5*

Figure S3.
Sequence of a region of the *TP53* gene, from exon 4 to exon 5. The exonic sequences are shown in red and intronic sequence in black fonts. The splice site mutations in IIICF/c and a breast cancer patient sample are shown in bold (in red and black fonts respectively) at the exon4/intron 4 boundary. These mutations result in an open reading frame that extends into intron 4, shown in black capitals, with an in-frame stop codon shown in italics. The purple lowercase font is the Δ133p53 5' untranslated sequence. Numbers in blue font refer to hg19 genome coordinates on chromosome 17.
